# Association of simultaneous sour taste stimulation with prefrontal cortex activation during stroop task performance in healthy adults: a fNIRS study

**DOI:** 10.3389/fnbeh.2026.1793337

**Published:** 2026-04-23

**Authors:** Xiao Juan Li, Ji Liang Kang, Fei Fei Ge, Tingting Ying, Shuang Liang Li, Yu Jin, Xiaobo Chen, Min Tang

**Affiliations:** 1 School of Rehabilitation, Gannan Medical University, Ganzhou, China; 2 Ningbo Rehabilitation Hospital, Ningbo, China; 3 Second Clinical Medical College, Shanxi Medical University, Taiyuan, China; 4 The Second Affiliated Hospital of Gannan Medical University, Ganzhou, China

**Keywords:** executive function, fNIRS, prefrontal cortex, sour taste stimulation, Stroop task

## Abstract

**Background:**

The prefrontal cortex (PFC) plays a central role in executive functions, particularly during cognitive conflict tasks. Sour taste is a salient oral sensory stimulus and may be associated with changes in behavioral performance and prefrontal activation during cognitive tasks. This study examined whether simultaneous sour taste stimulation was associated with behavioral and prefrontal hemodynamic changes during Stroop task performance in healthy adults using functional near-infrared spectroscopy (fNIRS).

**Methods:**

Twenty-six right-handed healthy adults were randomly assigned to either the sour taste group (0.1 M citric acid, *n* = 13) or the water control group (*n* = 13). Participants completed a simplified color-word Stroop task in a between-subject block design while receiving simultaneous oral stimulation through cotton swabs placed on the anterior-lateral surface of the tongue. A 52-channel fNIRS system recorded changes in oxygenated and deoxygenated hemoglobin concentrations in the frontal cortex, while behavioral data (reaction time, accuracy) were collected concurrently. Behavioral data were analyzed using mixed-design analysis of variance and interference-score comparisons. Task-related Oxy-Hb responses were analyzed using a general linear model with false discovery rate correction, whereas Deoxy-Hb signals were reviewed descriptively only.

**Results:**

The sour taste group showed faster overall reaction times and higher overall accuracy than the water control group during Stroop task performance. However, no significant between-group differences were observed in Stroop interference scores for reaction time or accuracy. At the neural level, both groups showed task-related frontal activation, while the sour taste group exhibited higher Oxy-Hb β values in several channels, particularly in the left dorsolateral prefrontal cortex and frontopolar region.

**Conclusion:**

Simultaneous sour taste stimulation was associated with better overall task performance and stronger task-related prefrontal hemodynamic responses in healthy adults during the Stroop task. These findings support an association between sour taste stimulation, broader behavioral facilitation, and altered prefrontal recruitment, but do not provide clear evidence for a conflict-specific enhancement of Stroop interference control.

## 1 Introduction

Executive functions (EF) play a central role in cognitive control processes (Skidmore et al., 2023), including attentional modulation, response inhibition, and conflict monitoring. The Stroop Color–Word Test (Stroop task) is one of the most widely used paradigms for assessing cognitive interference and executive control, and it is commonly used to evaluate performance under conditions of cognitive conflict (Periáñez et al., 2021). In this task, participants are required to respond to the font color while ignoring the semantic meaning of the word, making it a classic measure of selective attention and inhibitory control (Freund et al., 2021). In the congruent condition, word meaning and font color are consistent, whereas in the incongruent condition they are mismatched, requiring participants to suppress the dominant tendency to read the word and instead respond to the font color. The performance difference between these two conditions, commonly referred to as Stroop interference, is widely used as an index of conflict-related processing demand and executive control efficiency. This process relies heavily on the prefrontal cortex (PFC), particularly in relation to attentional control, response selection, and the management of cognitive conflict during task performance (Yeung et al., 2020).

External intervention methods, particularly sensory stimulation, have been shown to modulate performance during cognitive tasks (Churnin et al., 2019). For example, caffeine intake has been reported to improve performance on the Stroop task (Yuan et al., 2020). Taste, as a fundamental sensory modality, is also closely linked to emotional and attentional processing. Among the five basic tastes, sourness is often experienced as a sharp, biologically salient oral signal and may be associated with heightened alertness and attentional engagement (Rousmans et al., 2000). Sour stimulation was selected in the present study for both theoretical and practical reasons. From a theoretical perspective, a salient oral sensory input may influence task performance by modulating sensory salience, arousal, and task engagement. From an experimental perspective, citric acid provides a simple, safe, and easily standardized method for delivering reproducible oral stimulation during task performance. Thus, sour taste was used here as an exploratory candidate stimulus to examine whether a salient oral sensory input is associated with behavioral facilitation and altered prefrontal recruitment during executive-task performance. However, because the present study did not compare multiple taste modalities, the findings should not be interpreted as indicating a specific advantage of sour taste over other forms of gustatory stimulation.

Although the modulatory effects of sour taste on emotion and sensory processing have been increasingly discussed, its influence on cognitive conflict tasks, particularly in relation to PFC activation, remains insufficiently explored (Zhou and Tse, 2020). Investigating whether sour taste stimulation is associated with changes in behavioral performance and prefrontal hemodynamic responses during Stroop task performance may help clarify how oral sensory stimulation relates to executive-task processing. The PFC plays a central role in attentional control, response selection, and cognitive conflict management (Soar et al., 2016; Soluoku and Hyman, 2024), making it a theoretically relevant region for the present study.

With the advancement of neuroscience technologies, functional near-infrared spectroscopy (fNIRS) has gained widespread application in studying cortical activity as a non-invasive imaging method (Curtin et al., 2019; Hu et al., 2019). fNIRS measures task-related hemodynamic responses by detecting changes in the absorption of near-infrared light by oxygenated and deoxygenated hemoglobin in superficial cortical tissue. It therefore provides relative estimates of oxygenated hemoglobin and deoxygenated hemoglobin changes associated with neural activity through neurovascular coupling (Chen et al., 2020). Traditional neuroimaging techniques such as functional magnetic resonance imaging (fMRI) and positron emission tomography (PET) provide high spatial resolution, but their high cost, operational complexity, and restrictions on subjects make them less suitable for some experimental contexts (Ko et al., 2013). Compared to these methods, fNIRS offers advantages in portability, ecological validity, and tolerance of natural task performance (Coltheart, 2013; Pereira et al., 2023). Because near-infrared light is most sensitive to superficial cortical regions, fNIRS is particularly suitable for monitoring activity in the PFC. In addition, previous fNIRS studies have demonstrated the sensitivity of prefrontal hemodynamic responses to executive-task demands and cognitive conflict (Causse et al., 2017; Liu et al., 2024), making this method appropriate for examining whether simultaneous sour taste stimulation is associated with altered task-related cortical recruitment.

Therefore, this study employed fNIRS to investigate whether simultaneous sour taste stimulation is associated with changes in behavioral performance and prefrontal cortex activation during Stroop task performance in healthy adults.

## 2 Materials and methods

**FIGURE 1:**
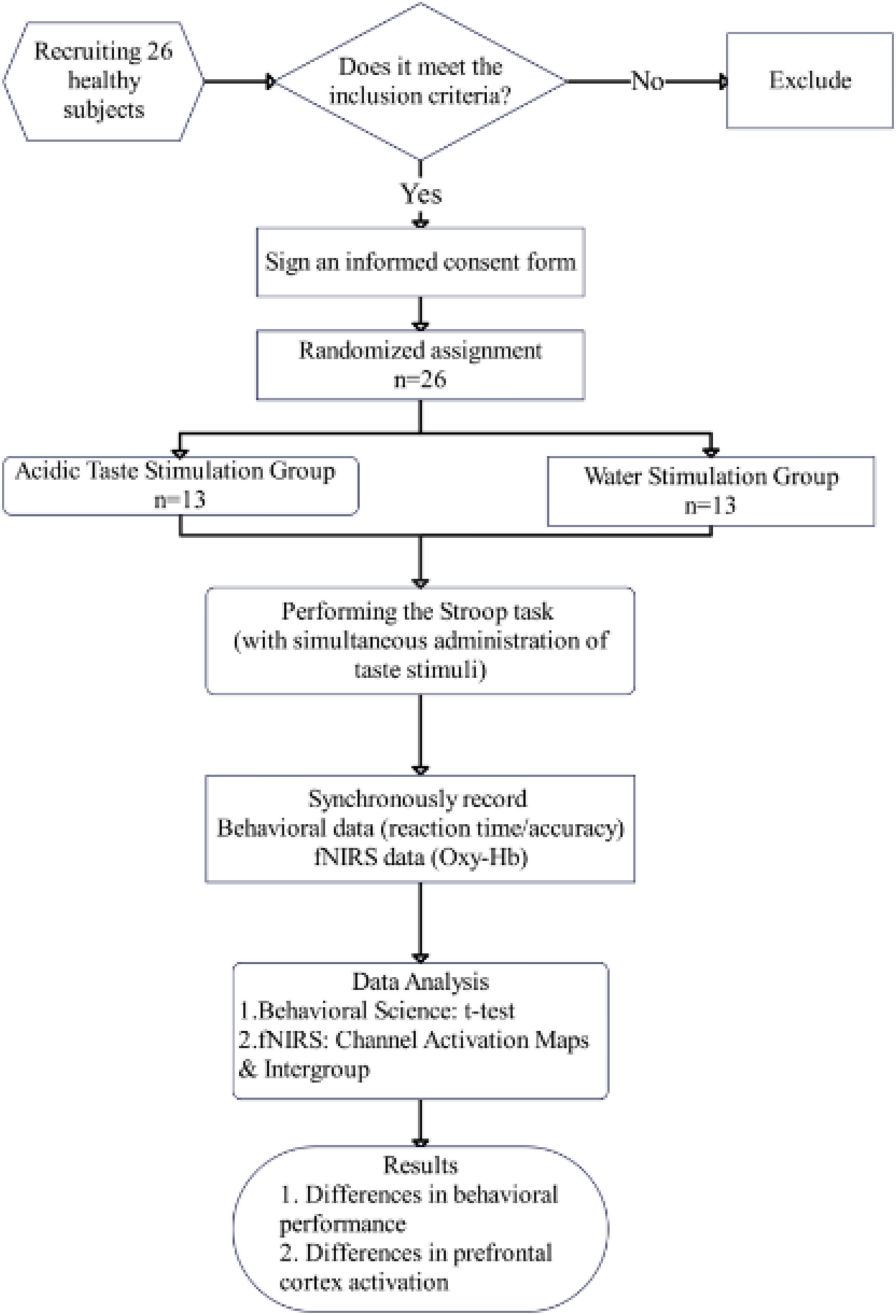
Experimental flowchart.

The present study employed a between-subjects experimental design. This was a preliminary exploratory study. The research process is shown in Figure 1. This study recruited 26 healthy adults (13 males, 13 females) from Ningbo Rehabilitation Hospital, all of whom were right-handed. Participants’ ages ranged from 20 to 25 years. The study was approved by the Ethics Committee of Ningbo Rehabilitation Hospital (approval number: 2025-35), and all participants signed informed consent forms and understood the experimental procedures.

### 2.1 Participants

Inclusion Criteria: (1) Participants must possess normal vision or corrected vision; (2) No color vision deficiency; (3) No history of neurological or psychiatric disorders; (4) No taste dysfunction; (5) No recent use of medications that may affect cognition or taste perception.

Participants were randomly allocated to the sour taste group (*n* = 13, age: 22.15 ± 1.63 years) or the water control group (*n* = 13, age: 22.54 ± 1.56 years) using a random-number table. There were no statistically significant between-group differences in age, sex distribution, or education level (all *p* > 0.05).

### 2.2 Research materials

The sour taste stimulation consisted of a 0.1 M citric acid solution prepared by dissolving food-grade citric acid in room-temperature purified water. The control stimulus was room-temperature purified water. Both solutions were administered using sterile cotton swabs.

### 2.3 Experimental design

This experiment employed a block design paradigm. The task was a simplified color-word Stroop task presented via E-Prime 2.0 (Won et al., 2019). Stimuli consisted of the Chinese characters for “red” and “blue” written in red or blue ink, forming congruent conditions (meaning matched color) and incongruent conditions (meaning conflicted with color). Each trial presented the stimulus for 5 s, requiring participants to ignore the word meaning and press the corresponding key as quickly as possible based on the font color (↑ for red, ↓ for blue). The response mapping was fixed across participants and was not counterbalanced.

Prior to the formal experiment, participants underwent a 5-min seated adaptation period and task practice to master the rules. The practice trials followed the same response rules as the formal task but were not included in the behavioral or fNIRS analyses.

**FIGURE 2:**
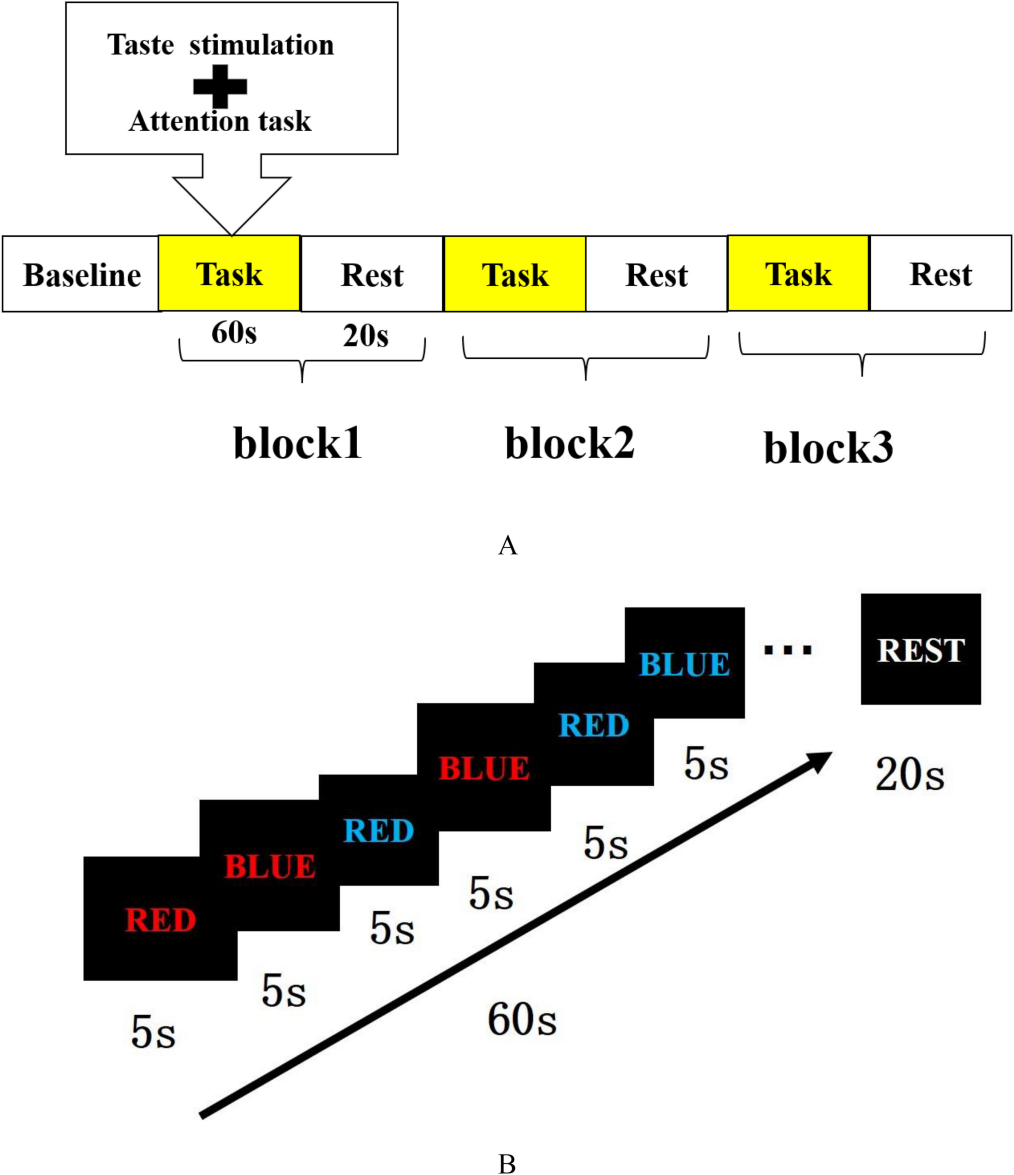
Experimental paradigms used in the present study. **(A)** Block design of the fNIRS experiment. Participants completed three 1-min Stroop task blocks separated by 20-s rest periods. Taste stimulation was administered during each task block and removed during the rest periods. **(B)** Simplified Stroop task paradigm. The Chinese characters for “red” and “blue” were presented in red or blue font, forming congruent and incongruent conditions. Participants were instructed to respond to the font color while ignoring the semantic meaning of the word.

The formal task comprised 3 blocks, each lasting 1 min and containing 12 trials (congruent and incongruent trials pseudo-randomly intermixed), with 20-s rest periods between blocks. Total task duration was approximately 4 min. The fNIRS task paradigm and the Stroop task paradigm are illustrated in Figure 2A,B, respectively.

Each trial began with the presentation of a color word stimulus and lasted 5 s in total. Participants were instructed to respond as quickly and accurately as possible according to the font color while ignoring the word meaning. Responses made within the 5 s window were recorded, but the stimulus remained on the screen until the end of the trial to maintain a fixed trial duration. The next trial started immediately after the previous 5 s interval ended, and no jittered inter-trial interval was used. Congruent and incongruent trials were pseudo-randomly intermixed within each block.

The taste stimuli were administered as follows: Before each task block commenced, the experimenter used a sterile cotton swab dipped in the corresponding solution (sour taste group: 0.1 M citric acid solution; water control group: purified water) and placed it on the anterior-lateral surface of the tongue. The swab remained in contact throughout the entire task block. During rest periods, the swab was removed so that taste stimulation was present only during the task blocks. Because the sensory properties of citric acid and water were readily distinguishable, participant blinding was not feasible. Experimenters administering the stimulation were also aware of group allocation. No formal manipulation check was conducted during the experiment (e.g., ratings of perceived sourness intensity, pleasantness/unpleasantness, discomfort, arousal, salivation, urge to swallow, or attentional distraction related to the tongue sensation).

### 2.4 fNIRS data acquisition

**FIGURE 3:**
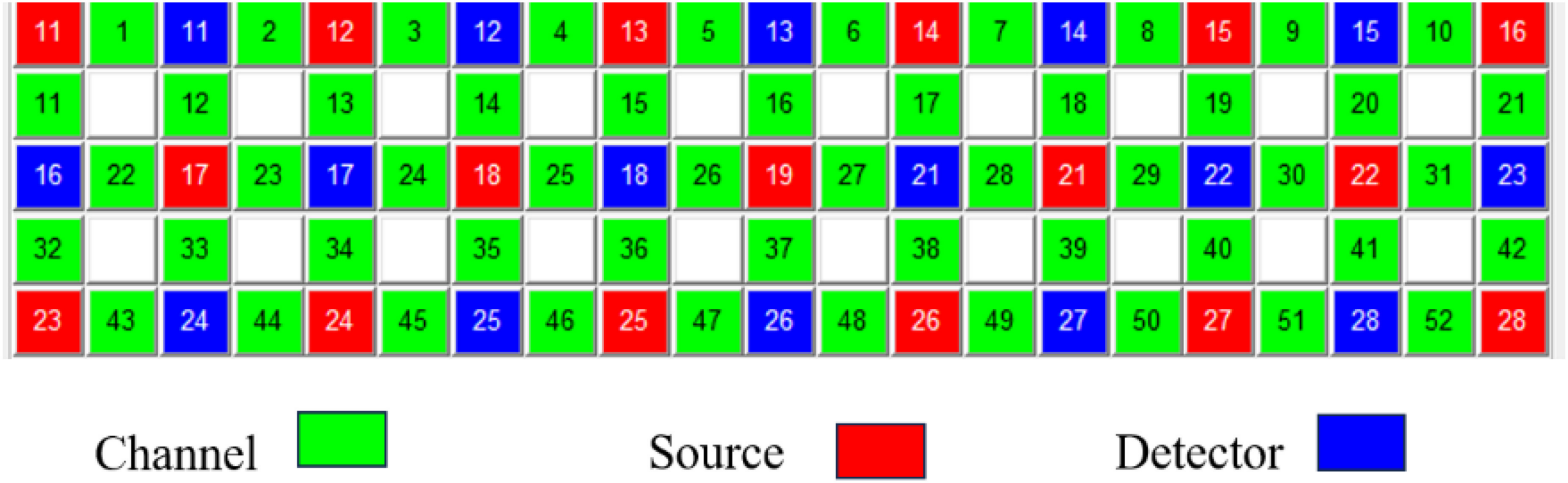
Probe arrangement and channel distribution of the 52-channel fNIRS system. The ETG-4100 probe set consisted of 17 light sources and 16 detectors arranged over the frontal scalp in a 3 × 11 matrix with an inter-optode distance of 3.0 cm, forming 52 measurement channels. The montage was positioned according to the international 10–20 system, with the midline aligned to Fpz, to cover frontal regions relevant to cognitive control. Anatomical labels represent approximate template-based localization.

This study employed a 52-channel fNIRS system (ETG-4100, Hitachi, Japan) to record cortical activity elicited by the Stroop task (Yang et al., 2023). The system uses scalp-mounted optodes consisting of light sources and detectors positioned over the frontal scalp. The system comprises 17 light sources and 16 detectors arranged in a 3 × 11 matrix over the frontal region, with a 3.0 cm spacing between light sources and detectors. The probe layout followed the international 10–20 system for positioning and was designed to cover frontal regions relevant to cognitive control, including the dorsolateral prefrontal cortex (DLPFC) and frontopolar area. Figure 3 shows the channel distribution of the fNIRS system. During probe positioning, the bottom row of detectors was aligned with the eyebrow line, and the midline channel corresponded to Fpz to ensure coverage of key frontal regions.

No participant-specific structural MRI was acquired in the present study. For anatomical visualization, cortical activation maps were generated in NIRS-KIT using a template-based brain rendering according to the standard probe arrangement referenced to the international 10–20 system. Therefore, the displayed anatomical locations represent approximate template-based projections for group-level interpretation rather than subject-specific cortical anatomy. Channel-to-region assignments reported in the tables were based on the manufacturer’s standard probe configuration and the corresponding anatomical labels derived from the 10 to 20 positioning framework; accordingly, these labels were used for regional description only.

The system operated at a sampling rate of 10 Hz. Each light source emits near-infrared light at 695 and 830 nm wavelengths. After passing through the scalp, skull, and superficial cortical tissue, the diffusely reflected light signals were detected by adjacent detectors. Based on a modified Beer-Lambert law, the system calculates real-time relative concentration changes of oxygenated hemoglobin (Oxy-Hb) and deoxygenated hemoglobin (Deoxy-Hb). Short-separation channels were not available in the current fNIRS setup. Therefore, systemic physiological artifacts could not be directly regressed out. Additionally, this study did not record physiological indicators such as heart rate or respiration.

### 2.5 Statistical analysis

#### 2.5.1 fNIRS data processing

After data acquisition, fNIRS data were processed and analyzed using the NIRS-KIT toolbox on the MATLAB platform (Hou et al., 2021). During preprocessing, raw hemoglobin concentration signals underwent first-order polynomial detrending to eliminate baseline drift, followed by motion artifact correction using the correlation-based signal improvement method (CBSI) (Cui et al., 2010). Subsequently, a 0.01–0.08 Hz band-pass filter was applied to suppress high-frequency physiological noise and instrument fluctuations while preserving low-frequency oscillations associated with neural activity (Li et al., 2022). Following preprocessing, task-related Oxy-Hb responses were estimated for each channel using a general linear model (GLM). All task blocks were modeled as a single task condition and convolved with a canonical hemodynamic response function (HRF), with the inter-block rest periods serving as the baseline condition. The contrast of interest was task versus rest. A β coefficient was obtained for each channel for each participant, reflecting the degree of task-related activation relative to baseline. These β values were subsequently incorporated into group-level statistical analyses. Within each group, one-sample *t*-tests against zero were performed on channel-wise β values to identify significant task-related activation relative to baseline, whereas between-group differences in channel-wise β values were assessed using independent-samples *t*-tests. Multiple comparisons across 52 channels were controlled using false discovery rate (FDR) correction, with a significance threshold set at FDR-corrected *p* < 0.05. Oxy-Hb was selected as the primary outcome measure because it is generally considered more sensitive to task-related cortical activation in fNIRS studies. Deoxy-Hb signals were reviewed as a secondary descriptive reference, but were not included in the primary channel-wise statistical inference.

#### 2.5.2 Processing of behavioral data

Behavioral data were automatically recorded by E-Prime 2.0, including reaction time (RT, in milliseconds) and response accuracy for each trial. Before statistical analysis, raw behavioral data from the sour taste group and water control group were exported and preprocessed. Accuracy (ACC) was calculated as the percentage of correct responses relative to total trials under each condition. Trials with no response within the 5 s response window were treated as omissions and counted as incorrect for accuracy analyses. For RT analyses, only correct-response trials were included, and anticipatory responses shorter than 200 ms were excluded.

After preprocessing, the mean reaction time and accuracy were calculated for each participant under congruent and incongruent conditions. Statistical analysis was performed using SPSS 27.0 software. A 2 × 2 mixed-design analysis of variance (ANOVA) was performed, with group (water control vs. sour taste) as the between-subjects factor and condition (congruent vs. incongruent) as the within-subjects factor. *Post hoc* comparisons using Bonferroni correction were conducted when significant interactions were observed. In addition to the mixed-design ANOVA, RT and ACC interference scores were compared between groups using independent-samples *t*-tests.

Stroop interference scores were calculated for RT and ACC. RT interference was defined as the difference between RT in the incongruent condition and RT in the congruent condition. ACC interference was defined as the difference between ACC in the incongruent condition and ACC in the congruent condition. All behavioral tests were two-tailed, with statistical significance set at *p* < 0.05.

## 3 Results

### 3.1 Behavioral findings: Stroop task

**FIGURE 4:**
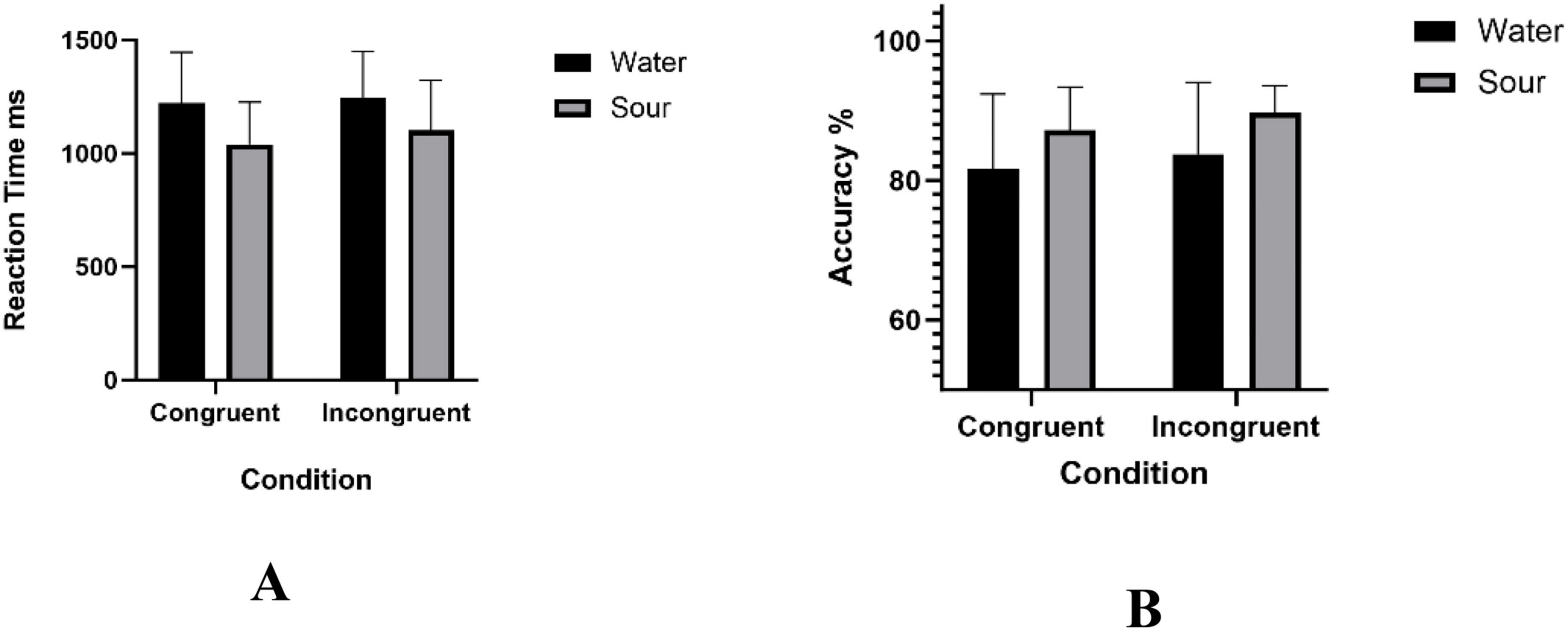
Behavioral performance in the Stroop task under water and sour taste stimulation. **(A)** shows reaction time and **(B)** shows accuracy under congruent and incongruent conditions in the water control group and sour taste group. Data are presented as mean ± SD. RT showed a significant main effect of group, *F*(1, 24) = 4.61, *p* = 0.042, and ACC also showed a significant main effect of group, *F*(1, 24) = 5.80, *p* = 0.024. No significant Group × Condition interaction was observed for RT or ACC.

Table 1 summarizes the behavioral results of the Stroop task, including RT and ACC under both congruent and incongruent conditions. Figure 4 presents the corresponding group comparisons for RT and ACC.

**TABLE 1:** Behavioral performance in the stroop task for the water and sour taste groups.

Measure	Condition	Water group (*n* = 13)	Sour group (*n* = 13)	Group effect	Condition effect	Group × Condition
RT (ms)	Congruent	1221.87 ± 225.58	1040.81 ± 185.98	*F*(1, 24) = 4.61	*F*(1, 24) = 1.79	*F*(1, 24) = 0.33
	Incongruent	1246.30 ± 203.94	1102.11 ± 220.38	*p* = 0.042*	*p* = 0.193	*p* = 0.570
	Interference	24.43 ± 193.77	61.30 ± 125.80	η^2^ *_*p*_* = 0.16	η^2^ *_*p*_* = 0.07	η^2^ *_*p*_* = 0.01
ACC (%)	Congruent	81.62 ± 10.73	87.18 ± 6.16	*F*(1, 24) = 5.80	F (1,24) = 1.16	*F*(1, 24) = 0.01
	Incongruent	83.76 ± 10.26	89.74 ± 3.83	*p* = 0.024*	*p* = 0.292	*p* = 0.923
	Interference	2.14 ± 14.62	2.56 ± 5.83	η^2^ *_*p*_* = 0.19	η^2^ *_*p*_* = 0.05	η^2^ *_*p*_* < 0.001

Data are presented as Mean ± Standard Deviation. RT, Reaction Time; ACC, Accuracy. Interference RT = RT _*Incongruent*_ − RT _*Congruent*_; Interference ACC = ACC _*Incongruent*_ − ACC _*Congruent*_. *Indicate statistically significant main effects of group at *p* < 0.05.

A 2 × 2 mixed-design ANOVA with group (water control group vs. sour taste group) as the between-subjects factor and condition (congruent vs. incongruent) as the within-subjects factor revealed a significant main effect of group on RT, F (1, 24) = 4.61, *p* = 0.042, η^2^*_*p*_* = 0.16, indicating that the sour taste group responded faster overall than the water control group. The main effect of condition was not significant, *F*(1, 24) = 1.79, *p* = 0.193, η^2^*_*p*_* = 0.07. The Group × Condition interaction was also not significant, *F*(1, 24) = 0.33, *p* = 0.570, η^2^*_*p*_* = 0.01. Specifically, mean RTs in the water control group and sour taste group were 1221.87 ± 225.58 ms and 1040.81 ± 185.98 ms, respectively, for congruent trials, and for incongruent trials were 1246.30 ± 203.94 ms and 1102.11 ± 220.38 ms. The RT interference score (RT _*incongruent*_ − RT _*congruent*_) was 24.43 ± 193.77 ms in the water control group and 61.30 ± 125.80 ms in the sour taste group. An independent-samples *t*-test showed that this between-group difference was not statistically significant, *t*(24) = −0.58, *p* = 0.570, Cohen’s *d* = −0.23.

For ACC, the mixed-design ANOVA revealed a significant main effect of group, *F*(1, 24) = 5.80, *p* = 0.024, η^2^*_*p*_* = 0.19, indicating that the sour taste group achieved higher overall accuracy than the water control group. The main effect of condition was not significant, *F*(1, 24) = 1.16, *p* = 0.292, η^2^*_*p*_* = 0.05. The Group × Condition interaction was also not significant, *F*(1, 24) = 0.01, *p* = 0.923, η^2^*_*p*_* < 0.001. In the congruent condition, ACC was 81.62% ± 10.73% in the water control group and 87.18% ± 6.16% in the sour taste group. In the incongruent condition, ACC was 83.76% ± 10.26% in the water control group and 89.74% ± 3.83% in the sour taste group. The ACC interference score (ACC _*incongruent*_ − ACC _*congruent*_) was 2.14% ± 14.62% in the water control group and 2.56% ± 5.83% in the sour taste group. The between-group difference was not statistically significant, *t*(24) = −0.10, *p* = 0.923, Cohen’s *d* = −0.04.

These results indicate that simultaneous sour taste stimulation was associated with better overall task performance, whereas no significant between-group differences were observed in Stroop interference scores.

### 3.2 fNIRS findings: brain activation patterns

#### 3.2.1 Brain activation patterns under water control stimulation

**FIGURE 5:**
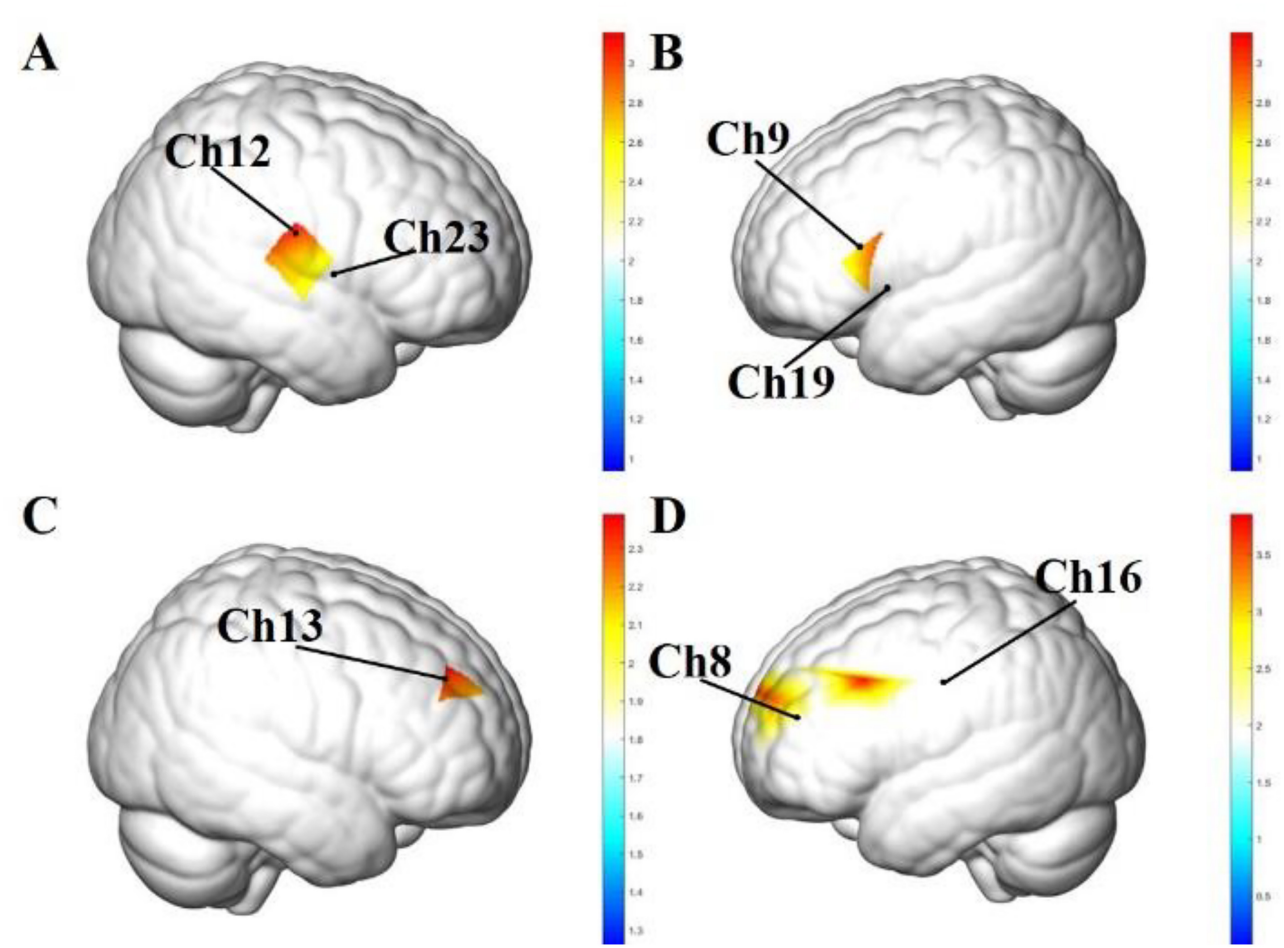
Significant task-related cortical activation maps during Stroop task performance in the water control and sour taste groups. **(A)** Water control group, right lateral view. **(B)** Water control group, left lateral view. **(C)** Sour taste group, right lateral view. **(D)** Sour taste group, left lateral view. The maps show channels with significant task-related increases in Oxy-Hb during Stroop task performance relative to rest within each group. Representative channels from Tables 2, 3 are approximately indicated on the template brain according to the standard probe arrangement referenced to the international 10–20 system, to facilitate correspondence between the activation maps and the tabulated results. The color bar represents statistical values (Stat), with warmer colors indicating larger values. Anatomical locations and channel labels represent approximate template-based projections rather than subject-specific cortical anatomy.

During the Stroop task under water stimulation, significant task-related Oxy-Hb activation relative to baseline was observed in channels 9, 12, 19, 22, 23, 29, 33, and 40. These channels were distributed across multiple frontal regions, including the left and right DLPFC, the pre-motor and supplementary motor cortex, the primary motor cortex, the pars opercularis, and the pars triangularis. Exact channel numbers, regional labels, β values, and corrected *p*-values are summarized in Table 2, and their spatial distribution is shown in Figure 5A,B, in which representative channels are annotated for visual reference.

**TABLE 2:** β values and *p* values during the Stroop task under water stimulation.

Channel	S-D	Brain area	β -value	*p*-value
9	S15-D15	Left dorsolateral prefrontal cortex	0.015 ± 0.014	0.002*
12	S17-D11	Pre-motor and supplementary motor cortex	0.018 ± 0.012	0.030*
19	S15-D22	Left dorsolateral prefrontal cortex	0.022 ± 0.022	0.040*
22	S17-D16	Primary motor cortex	0.02 ± 0.03	0.032*
23	S17-D17	Right dorsolateral prefrontal cortex	0.011 ± 0.031	0.010*
29	S21-D22	Left dorsolateral prefrontal cortex	0.012 ± 0.022	0.025*
33	S17-D24	Pars opercularis	0.011 ± 0.031	0.028*
40	S27-D22	Pars triangularis	0.018 ± 0.032	0.020*

Values are presented as the mean ± standard deviation. Channels listed showed significant task-related activation relative to baseline after FDR correction (*p < 0.05 after FDR correction). S-D, source-detector. *P*-values are FDR-corrected channel-wise one-sample *t*-tests of participant-level β coefficients against zero.

#### 3.2.2 Brain activation patterns under sour taste stimulation

During the Stroop task under sour taste stimulation, significant task-related Oxy-Hb activation relative to baseline was observed in channels 4, 8, 13, 16, 17, 23, and 46. These channels were distributed across the left DLPFC, right DLPFC, frontopolar area, and frontal eye field. Exact channel numbers, regional labels, β values, and corrected *p*-values are summarized in Table 3, and their spatial distribution is shown in Figure 5C,D, in which representative channels are annotated for visual reference.

**TABLE 3:** β values and *p*-values during the Stroop task under sour taste stimulation.

Channel	S-D	Brain area	β -value	*p*-value
4	S13-D12	Frontal eye field	0.012 ± 0.018	0.034*
8	S15-D14	Left dorsolateral prefrontal cortex	0.015 ± 0.014	0.002*
13	S12-D17	Right dorsolateral prefrontal cortex	0.01 ± 0.023	0.015*
16	S19-D13	Left dorsolateral prefrontal cortex	0.01 ± 0.01	0.005*
17	S14-D21	Left dorsolateral prefrontal cortex	0.01 ± 0.011	0.010*
23	S17-D17	Right dorsolateral prefrontal cortex	0.031 ± 0.009	0.024*
46	S25-D25	Frontopolar area	0.019 ± 0.013	0.036*

Values are presented as the mean ± standard deviation. Channels listed showed significant task-related activation relative to baseline after FDR correction (*p < 0.05 after FDR correction). S-D, source-detector. *P*-values are FDR-corrected channel-wise one-sample *t*-tests of participant-level β coefficients against zero.

#### 3.2.3 Intergroup brain activation patterns

**FIGURE 6:**
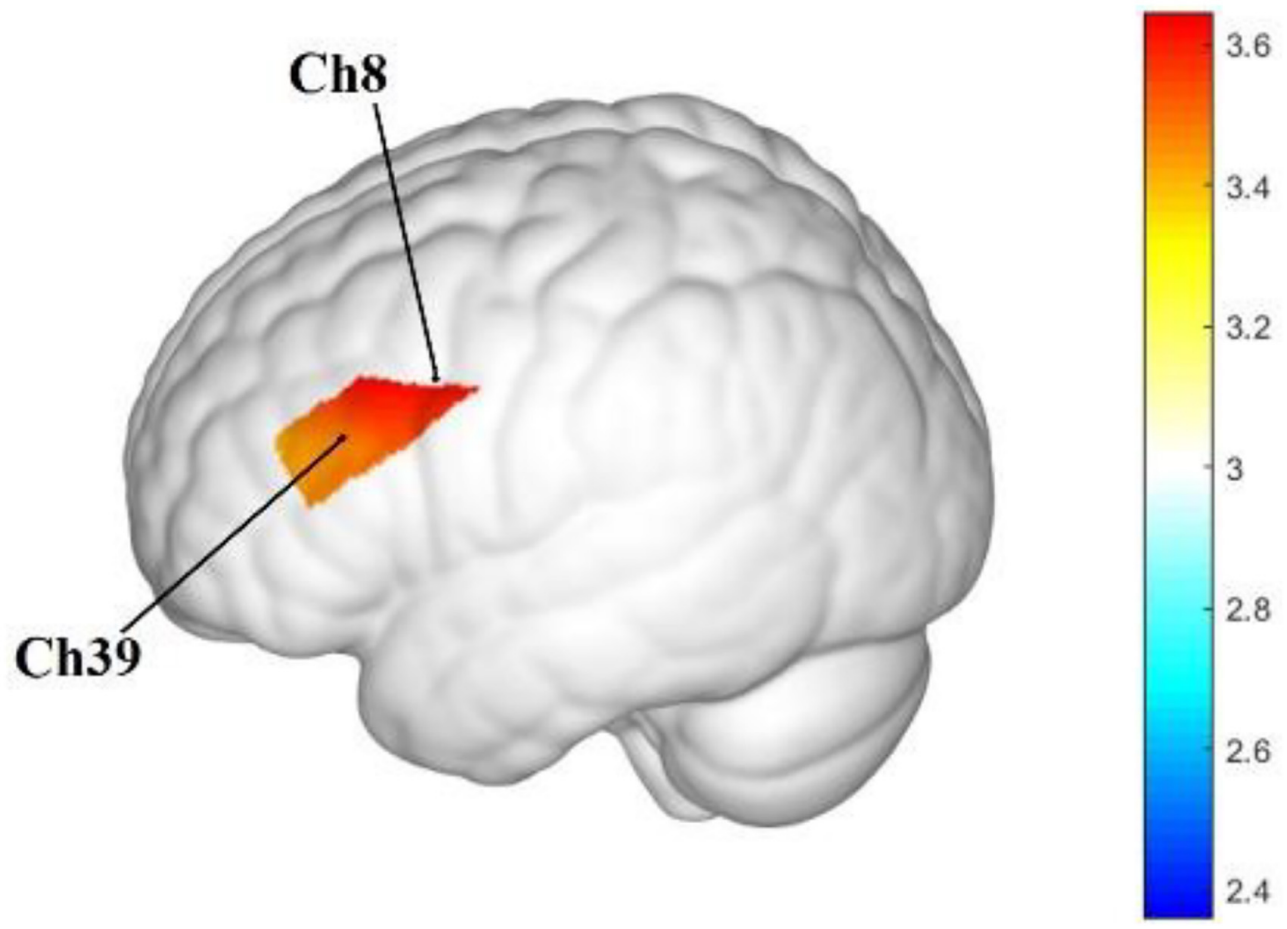
Significant between-group differences in task-related cortical activation during Stroop task performance. The map shows channels with significantly higher task-related Oxy-Hb responses in the sour taste group than in the water control group. Because the significant between-group differences were mainly located in the left frontal cortex, only the left lateral view is shown. Representative channels from Table 4 are approximately indicated on the template brain according to the standard probe arrangement referenced to the international 10–20 system, to facilitate correspondence between the activation map and the tabulated results. The color bar represents statistical values (Stat), with warmer colors indicating larger values. Anatomical locations and channel labels represent approximate template-based projections rather than subject-specific cortical anatomy.

Between-group comparison of channel-wise β values identified significant differences in channels 8, 9, 16, 18, 19, 29, and 39. These channels were mainly located in the left DLPFC, including channels 8, 9, 16, 18, 19, and 29, whereas channel 39 corresponded to the frontopolar area. In these channels, β values were higher in the sour taste group than in the water control group. Exact channel numbers, regional labels, β values, and corrected *p* values are reported in Table 4, and the spatial distribution of the significant between-group differences is shown in Figure 6, in which representative channels are annotated for visual reference.

**TABLE 4:** Comparison of β values during Stroop task performance between the water control group and the sour taste group.

Channel	S-D	Brain area	Water	Sour	*p*-value
8	S15-D14	Left dorsolateral prefrontal cortex	0.002 ± 0.003	0.015 ± 0.014	0.002*
9	S15-D15	Left dorsolateral prefrontal cortex	0.015 ± 0.014	0.02 ± 0.02	0.002*
16	S19-D13	Left dorsolateral prefrontal cortex	0.003 ± 0.009	0.01 ± 0.01	0.021*
18	S21-D14	Left dorsolateral prefrontal cortex	0.02 ± 0.02	0.04 ± 0.02	0.029*
19	S15-D22	Left dorsolateral prefrontal cortex	0.022 ± 0.022	0.028 ± 0.023	0.009*
29	S21-D22	Left dorsolateral prefrontal cortex	0.012 ± 0.022	0.015 ± 0.022	0.018*
39	S21-D27	Frontopolar area	0.016 ± 0.013	0.025 ± 0.015	0.012*

Data are presented as Mean ± Standard Deviation. Channels listed showed significant between-group differences after FDR correction (*p < 0.05 after FDR correction). *P*-values are FDR-corrected channel-wise independent-samples *t*-tests comparing participant-level β coefficients between groups.

## 4 Discussion

This study used fNIRS to examine behavioral performance and prefrontal hemodynamic responses during Stroop task performance under simultaneous sour taste stimulation in healthy adults. The sour taste group showed faster responses and higher accuracy than the water control group. At the same time, the magnitude of Stroop interference did not differ significantly between groups. At the neural level, the sour taste group showed higher task-related Oxy-Hb responses in several prefrontal channels, with the most prominent differences located in the left dorsolateral prefrontal cortex and the frontopolar region. Taken together, these findings suggest that sour taste stimulation was associated with better overall task performance and stronger prefrontal recruitment, but did not provide clear evidence for a conflict-specific enhancement of Stroop interference control.

The behavioral pattern observed here is more consistent with a general facilitation effect than with a specific reduction in cognitive conflict. Participants in the sour taste group responded more quickly and more accurately across the task as a whole, yet the difference between congruent and incongruent conditions was not significantly altered. This interpretation is broadly compatible with the classic account of Stroop interference described by MacLeod and MacDonald (2000) and also resembles the overall performance facilitation reported in arousal-related manipulations such as caffeine administration by Yuan et al. (2020). A reasonable explanation is that sour taste increased oral sensory salience, heightened alertness, and strengthened task engagement. Under this condition, participants may have maintained the task rule more effectively across the entire testing period, which would improve overall speed and accuracy without necessarily reducing Stroop interference.

An additional point requiring caution is that the present simplified Stroop paradigm did not produce a robust classical interference pattern at the group level, as neither the main effect of condition nor the Group × Condition interaction reached significance for RT or ACC. Therefore, the task in the current study may have indexed executive-task engagement more broadly rather than strong conflict-specific processing.

A more detailed neural account can be considered from the perspective of sensory-arousal modulation. Sourness is a biologically salient oral signal. It is not processed only as a taste, but is often experienced as a sharp and attention-capturing oral sensation. This kind of stimulation may recruit gustatory, somatosensory, and trigeminal-related afferent processing at the same time. Current work on sour taste mechanisms has emphasized the complexity of peripheral and central pathways involved in sour signal transmission (Zhang et al., 2022). Chen et al. (2023) also reported that taste exposure was associated with altered resting-state low-frequency fluctuation in regions including frontal areas, while Kaskan et al. (2019) showed that gustatory stimulation can engage distributed cortical systems beyond primary taste processing areas. Seen together, these findings support the possibility that sour stimulation can influence brain state at a broader systems level. The present results are broadly consistent with this interpretation, although the underlying mechanism cannot be established in the current design. Sour stimulation may have placed the brain in a more behaviorally ready state, increased attentional mobilization, and improved response readiness during task execution. This framework helps explain why the sour taste group showed better overall performance, while the interference component itself remained unchanged.

A further possible explanation involves partial convergence of different forms of oral sensory stimulation on central arousal-related neuromodulatory systems. Recent work on oral mechanical stimulation has suggested that chewing-related cognitive facilitation may be mediated, at least in part, through trigeminal influences on the ascending reticular activating system, with the locus coeruleus–noradrenergic system considered a plausible candidate pathway (Tramonti Fantozzi et al., 2021). Although the primary peripheral afferent route of sour taste stimulation differs from that of oral mechanical stimulation, sour stimulation is not necessarily restricted to a purely gustatory channel and may also recruit somatosensory and trigeminal-related oral sensory components (Tramonti Fantozzi et al., 2021; Zhang et al., 2022). From this perspective, it is plausible that salient sour stimulation enhanced attentional readiness and task engagement through partially overlapping central arousal mechanisms, which could in turn contribute to stronger recruitment of prefrontal control-related regions such as the left dorsolateral prefrontal cortex and the frontopolar area. However, this interpretation remains speculative in the present study, because no direct measures of locus coeruleus activity, pupillometry, autonomic arousal, or brainstem responses were obtained.

The fNIRS findings are consistent with this broader interpretation. Both groups showed task-related activation in frontal regions commonly linked to cognitive control. Compared with the water control group, the sour taste group showed higher β values in several channels, especially in the left DLPFC and the frontopolar region. The DLPFC is widely regarded as a core region for Stroop-related control and the suppression of distracting input (Ehlis et al., 2024). Additional work has also linked prefrontal activation to arousal-related improvements in executive performance (Byun et al., 2014) and has shown that direct modulation of the left DLPFC can influence Stroop conflict resolution (Parris et al., 2021). Against this background, stronger Oxy-Hb responses in the sour taste group are better understood as reflecting greater recruitment of prefrontal task-control systems during task execution.

The left DLPFC may be especially relevant in this context. During Stroop task performance, this region is closely related to top-down control over stimulus selection and response mapping (Parris et al., 2021; Ehlis et al., 2024). If sour taste increased sensory salience and general alertness, then the left DLPFC may have been recruited more strongly to stabilize the rule of responding to color rather than word meaning. This would improve overall behavioral efficiency and would also be compatible with the absence of a significant group difference in interference scores. The frontopolar result adds another layer to this account. In the present context, stronger activation in the frontopolar region may reflect a more sustained control state during task performance. This may indicate that sour stimulation influenced not only immediate attentional focus, but also the broader cognitive mode required to remain engaged with the task.

Several limitations should be acknowledged. The between-subject design and relatively small sample size increase susceptibility to inter-individual variability in baseline cognitive performance, taste sensitivity, and arousal state, and no baseline Stroop assessment was obtained before stimulation. The fixed response mapping and limited number of blocks and trials may also have reduced the sensitivity of the simplified Stroop paradigm to detect a stable interference effect. Participant blinding was not feasible because citric acid and water were readily distinguishable, and experimenters were also aware of group allocation, so expectancy-related influences cannot be excluded. In addition, water served as a neutral comparator rather than an intensity-matched placebo, and no formal manipulation check or subjective ratings of sourness intensity, pleasantness, discomfort-driven vigilance, arousal, salivation, urge to swallow, or attentional anchoring on the tongue sensation were collected. Short-separation channels and physiological regressors were unavailable, and heart rate and respiration were not recorded, so systemic physiological contributions to the Oxy-Hb responses cannot be fully ruled out. In addition, Deoxy-Hb was not included in the formal channel-wise statistical reporting, which limits the hemodynamic completeness of the present findings. Finally, behavioral and fNIRS measures were analyzed separately, and direct brain-behavior associations were not examined. Future studies should adopt within-subject crossover designs, include baseline and manipulation assessments, collect subjective and physiological measures, and further clarify whether sour stimulation influences conflict-specific processing or a broader arousal-related facilitation of task performance.

## 5 Conclusion

This study showed that simultaneous sour taste stimulation was associated with faster overall responses, higher overall accuracy, and stronger task-related Oxy-Hb responses in several prefrontal channels during Stroop task performance in healthy adults. The main neural differences were observed in the left dorsolateral prefrontal cortex and frontopolar region. However, because no significant between-group differences were found in Stroop interference scores, the present findings do not provide clear evidence that sour taste selectively enhanced conflict-specific processing. A more cautious interpretation is that sour taste stimulation was associated with broader facilitation of task performance and altered prefrontal activation during task execution. These findings may provide a basis for further research on how oral sensory stimulation relates to executive-task performance, but the underlying mechanisms require confirmation in larger and more rigorously controlled studies.

## Data Availability

The raw data supporting the conclusions of this article will be made available by the authors, without undue reservation.
